# Incidence of genital warts among the Hong Kong general adult population

**DOI:** 10.1186/1471-2334-10-272

**Published:** 2010-09-17

**Authors:** Chunqing Lin, Joseph TF Lau, King-Man Ho, Man-Chun Lau, Hi-Yi Tsui, Kuen-Kong Lo

**Affiliations:** 1 Centre for Epidemiology and Biostatistics, the Chinese University of Hong Kong, Room 504, School of Public Health and Primary Care, Shatin, New Territories, SAR Hong Kong; 2 Centre for Medical Anthropology and Behavioural Health, School of Sociology and Anthropology, Sun Yat-sen University, 135, Xin Gang Xi Road, Guangzhou, 510275, China; 3 Social Hygiene Service Headquarter, the Public Health Service Branch, Center for Health Protection, Department of Health, Cheung Sha Wan Dermatological Clinic, 3/F West Kowloon Health Centre, 303 Cheung Sha Wan Road, Kowloon, SAR Hong Kong

## Abstract

**Background:**

The objective of this study is to estimate the incidence of genital warts in Hong Kong and explore a way to establish a surveillance system for genital warts among the Hong Kong general population.

**Methods:**

A total of 170 private doctors and all doctors working in the 5 local Social Hygiene Clinics (SHC) participated in this study. During the 14-day data collection period (January 5 through18, 2009), the participating doctors filled out a log-form on a daily basis to record the number of patients with genital warts. The total number of new cases of genital warts presented to private and public doctors in Hong Kong was projected using the stratification sampling method.

**Results:**

A total of 721 (0.94%) adults presented with genital warts to the participating doctors during the two-week study period, amongst them 73 (10.1%) were new cases. The projected number of new cases of genital warts among Hong Kong adults was 442 (297 male and 144 female) during the study period. The incidence of genital warts in Hong Kong was estimated to be 203.7 per 100,000 person-years (respectively 292.2 and 124.9 per 100,000 person-years for males and females).

**Conclusions:**

The incidence of genital warts is high among adults in Hong Kong. The study demonstrates the importance of collecting surveillance data from both private and public sectors.

## Background

Genital warts (condylomata acuminata) are highly infectious benign epithelial mucosal tumors caused by human papillomavirus (HPV), most commonly types 6 and 11[1,2]. Clinical symptoms of genital warts include itching, burning, tenderness at the warts site and anal, urethral, or vaginal bleeding or discharge[3]. Although not life threatening, genital warts exact a significant financial toll on the health care system and pervasive psychological stress and shame among the patients[4]. Genital warts is one of the most commonly occurring sexually transmitted diseases (STDs). The prevalence of genital warts has been estimated to be 1% in the sexually active population[5]. There prevalence of the diseases has been increasing throughout Europe[6,7]. In the United Kingdom, the prevalence of genital warts increased by approximately 25% from 1996 to 2005[8].

Surveillance of genital warts as well as other STDs should provide accurate and timely population estimates on prevalence and incidence trends[9]. Most of the STD surveillance studies have focused only on some specific high risk groups [10] as population-based surveys are difficult to implement[11]. In many countries, case reporting remains the mainstay of STD surveillance and very limited data are obtained from private patients[12]. In Hong Kong, surveillance data on STDs are solely obtained from the Social Hygiene Clinics (SHC) of the Department of Health. A surveillance study reported a total of 2,276 genital wart cases in Hong Kong in 2008[13]. The number and proportion of genital wart cases being diagnosed by local private doctors remain unknown. In addition, the incidence of genital warts was not documented in Hong Kong.

This study investigated the total number of new genital wart cases among patients who sought consultations from three types of private doctors, as well as public doctors working in the SHC, during a 14-day study period. The total number of new genital wart cases presenting their symptoms to Hong Kong doctors during the study period was projected. The objective of the study was to estimate the incidence of genital warts among general adult population in Hong Kong. A secondary objective of the study was to examine the feasibility of setting up a surveillance system for genital warts among the Hong Kong general population.

## Methods

### Sampling frame

In Hong Kong, about 56% of the physicians work in the private sector. The private system provides 20% of the inpatient medical care, 11% of hospital beds, and 70% of the outpatient visits. Patients generally seek private outpatient medical care by self-referral. Medical fees are usually paid at the time of service, with less than 10% of fees being paid directly by insurance companies[14]. Three types of private practitioners participated in the study: general practitioners (GP), obstetric and gynaecology (O&G) doctors and dermatologists and venereology (D&V) doctors. The majority of the local patients seeking consultation for STDs from private doctors would be seen by such private doctors[15,16]. A previous study which was commissioned by the Department of Health in Hong Kong in June 2007 requested the aforementioned types of private doctors and public doctors to record information of all patients presenting with STD symptoms within a specific period of 15 days[17]. In that study, the sampling frame of the private doctors included all members of the Hong Kong Association of Specialists in Dermatology and all O&G and GP doctors whose valid contact information was listed in the websites of the Medical Council of Hong Kong http://www.mchk.org.hk/doctor/index.htm, the Yellow Pages http://www.yp.com.hk/home/en/html/main/home.aspx and The Hong Kong Doctors Homepage http://www.hkdoctors.org of the Hong Kong Medical Association. Some research assistants then verified the validity of the contact information by phone or by mail. A total of 277 private doctors (247 GPs, 14 O&G doctors and 16 D&V doctors) participated in the 2007 study (representing 9.3%, 7.9% and 30.2% of all doctors with valid contact information) and all of them were invited to participate in the present study.

There are a total of eight Social Hygiene Clinics (SHC) in Hong Kong which are responsible for the prevention and control of STDs. SHC provide walk-in service for consultation, counselling and medical treatment for patients with STDs. Staff of the SHC carry out contact tracing, health education and outreach activities to control the spread of STDs[18]. In collaboration with the Department of Health, all doctors in five major SHC, out of the eight clinics, participated in the present study.

### Recruitment procedure

An invitation package which included a self-explanatory introduction letter, a sample log-form and a reply slip was sent to each of the aforementioned 277 doctors from November to December 2008. All prospective participants were requested to fill out the reply slip and return it to the research team by fax or mail. About one week after the invitation letter was sent, a follow-up telephone call was made to all prospective participants by a team of experienced interviewers to confirm their participation status. With respect to unanswered calls, at least four other independent calls were made on different hours in different days before the contact number was considered as an invalid one.

### Data collection

A study kit, which contained an instruction manual, a set of log forms and a reminder sheet, was delivered to the participants' clinic by a research assistant one week prior to the commencement of the data collection time period. The instruction manual introduced the objective of the study and the data collection procedure in detail to the doctors. Doctors in the SHS clinics received training on how to fill out the log-form. An enquiry hot-line was also launched during the study period. The anonymous log-form was designed by the research team to record the number of patients seen by the doctor on a particular day and the number of adult patients (aged 18 or above) presenting with genital warts to the doctor. The patients' names or other personal identifiable information were not collected. Genital warts were defined as clinical features of warts in or around anogenital area which include internal/external genitalia, perineum and perianal region. Diagnosis of genital warts is primarily based on clinical judgments and no laboratory tests were required. The patients with genital warts were asked whether he/she had ever noted similar symptoms before or been diagnosed with genital warts previously in any health care facilities. The cases with no past symptoms or clinical diagnosis of genital warts were defined as new cases. Demographic information such as gender and year of birth of the genital wart cases was recorded.

During the 14-day data collection period (January 5 to January 18, 2009), all participating doctors were required to fill out the log-form on a daily basis and fax it back to the research office. If the log-form was not received from a particular clinic on time, reminder calls were made by the research team to ensure the completeness of data. Long sensitive questionnaires were avoided in order to ensure the data accuracy. Research assistants cross-checked every form on a daily basis and contacted the doctor involved in the case of any unclear entries. The completed log forms were sent to the research team at the end of the data collection period for verification. As an incentive, a supermarket coupon (HK$200 or about US$25) was sent to participating doctors who completed the study. Ethics approval was obtained from the Chinese University of Hong Kong.

### Data analysis

The total number of patients presenting with genital warts to local private doctors during the data collection period was projected using the stratification sampling method[19,20]. GPs were stratified in 18 strata according to the district-board classification system. Hence, there were a total of 20 strata (18 GP strata + 1 O&G doctor stratum + 1 D&V doctor stratum) for private doctors. The total numbers of GPs in each of the 18 districts and the number of D&V and O&G were obtained from the Department of Health. The projected number of patients in a stratum is hence:

N=Total Number of doctor in the stratumNumber of participating doctor in the stratum×N'

N: projected number of patients in the stratum

N': Observed number of patients in the stratum

The territory-wide number of patients presenting with genital warts to private doctors during the study period was estimated by summing up the figures projected for all the 20 strata.

The number of adult genital warts patients seen by doctors of the five SHC during the study period was used to project the total number of adult patients seen by doctors of all eight SHC in the territory, according to the distribution of the number of genital warts cases presented in the eight individual clinics identified from a previous STD surveillance study conducted in 2007[17]. Such data were obtained from the local Department of Health. An important assumption was made that the number of genital warts patients seen by the participating doctors was representative of those seen by all doctors territory-wide.

The total number of new adult genital warts cases in Hong Kong presenting with symptoms to local private and public doctors during the study period was obtained by summing up the projected number of patients seen by private and public doctors. Bootstrap methods were used to estimate the respective 95% confidence intervals[21]. The intervals were calculated using Matlab V7.1 (the MathWorks, Inc.), based on a reasonably large number of 30000 bootstrap replications.

The incidence of genital warts was estimated using the total projected number of new genital warts cases detected by public and/or private doctors in Hong Kong during the study period, divided by the person-year exposure of the Hong Kong adult population age 18 and above during the same time period. According to the 2006 census, the sizes of the local male, female and total adult population age 18 and above years old were respectively 2,650,340, 3,006,691 and 5,657,031. The person-year exposure to the risk of developing new genital warts during the study period hence equals to the adult population size multiplied by 14/365.

## Results

### Participating doctors

A total of 215 GPs, 14 O&G doctors and 16 D&V doctors with valid contact information were invited to join the study and 157 GPs, seven O&G doctors and six D&V doctors participated in the study; the response rates were hence 73.0%, 50.0% and 37.5% for the three type of private doctors. Amongst the 170 participating private doctors, 140 (82.4%) were male. During the study period, a total of 76,060 patients visited the participating private and public doctors, amongst whom 73,036 were seen by private doctors and 3,024 were seen by public doctors (Table 1).

**Table 1:** Number of patients presenting the genital warts symptoms in the studied clinics in Hong Kong during the study period (January 5 to January 18, 2009)

Type of doctor (Number participated/number contacted)	Total number of patients	Number of genital wart cases	Number of new genital wart cases
		N (%) *	N (%) *
Private doctors	73,036	96 (0.13)	27 (0.04)
GP (157/215)	69,671	64 (0.09)	17 (0.02)
O&G doctor (7/14)	770	16 (2.08)	0 (0.000)
D&V doctors (6/16)	2,595	16 (0.62)	10 (0.39)
Public doctors	3,024	625 (20.67)	46 (1.52)
Total	76,060	721 (0.95)	73 (0.10)

* The percentages of the genital warts symptoms were calculated by the number of patients with the genital wart symptoms divided by the total number of patients who sought medical care during the study period.

### Profile of the genital warts cases

Amongst all patients visiting the participating doctors (76,060), 721 (0.94%) presented with genital warts during the study period. The majority (82.0%) of them were male. Approximately one tenth (73) of the 721 episodes were new cases. Amongst these new cases, 27 (37%) were identified by private doctors and 46 (63%) by public doctors (public: private ratio = 1.70: 1). Amongst the 27 patients who were identified by private doctors, 17 were identified by GPs and 10 by D&V doctors. The majority (79.5%) of the new cases were male (Table 1). About one third (31.5%) of them were older than 50 and 28.8% were between 18 and 30 years of age. Comparing new genital warts cases identified by private or public doctors, no statistically significant differences in age and gender were found (Table 2).

**Table 2:** The gender and age distributions of patients presenting new genital warts symptoms in the public sector versus the private sector in Hong Kong (2009)

	All (N = 73)	Private* (N = 27)	Public^# ^(N = 46)	χ^2^
		N (%)	N (%)	p-value
Gender				
Male	58	21 (78)	37 (80)	0.79
Female	15	6 (22)	9 (20)	
Age group				
<18	0	0 (0)	0 (0)	0.07
18-30	21	4 (15)	17 (37)	
31-40	19	11(41)	8 (17.)	
41-50	9	4 (15)	5 (11)	
>50	23	7 (26)	16 (35)	
Missing	1	1 (4)	0 (0)	

* Includes those patients who sought medical care in the private sector: General Practices, Obstetrics & Gynaecology and Dermatology & Venereology

# Includes those patients who sought medical care in the SHS clinics of the Department of Health

### Projected territory-wide number and incidence of new genital wart cases

The number of Hong Kong adults (18 or above years old) who presented with new genital warts to all local doctors during the study period was projected - 384 (95% CI: 208 to 590) for private patients and 58 (95% CI: 53 to 96) for public patients (private to public ratio = 6.62:1). Only respectively 15.4% and 7.6% of the projected male and female new genital warts cases were detected by public doctors (Table 3). The incidence of genital warts detected by either public or private doctors for the entire Hong Kong adult population is estimated to be 203.70 per 100,000 person-years (292.16 and 124.86 per 100,000 person-years respectively for males and females; Table 3).

**Table 3:** Projected number of patients presenting new genital wart symptoms and incidence among Hong Kong adults (2009)

	Observed cases	Projected Number of cases	**Person-year**^**1**^	**Incidence**^**2**^
Male				
Private	21	251		246.91
Public	37	46	101,657	45.25
All	58	297		292.19
				
Female				
Private	6	133		115.33
Public	9	11	115,325	9.54
All	15	144		124.86
				
All				
Private	27	384		176.97
Public	46	58	216,982	26.73
All	73	442		203.70

1. Person-year exposure (person-year) among those aged 18 or above = (total number of persons aged 18 or above in HK) * (14 days/365 days), where the total number of persons aged 18 or above in HK for male, female and all were respectively 2,650,340, 3,006,691 and 5,657,031 (source by 2006 population by-census).

2. Incidence rate (per 100,000 person-year) of new genital warts presented to doctors among Hong Kong adults aged 18 or above = (Projected no. of new cases)/(Person-year exposure (person-year))*100,000

## Discussion

The estimated incidence of genital warts was around 203.7 per 100,000 person-years, which is higher than those obtained from a number of international studies. A study reviewed the medical records obtained from 899 gynaecologists, dermatologists and urologists in Spain and provided an estimated incidence of 118 per 100,000 person-years[22]. In Canada, the incidence of genital warts was estimated to be 107 per 100,000 person-years in 1999, which increased slightly to 126 per 100,000 person-years in 2006[23]. In Germany, the incidence of genital warts was 113.7 per 100,000 person-years, based on the data collected from some gynecologists, dermatologists, and urologists seeing patients with genital warts[24]. The data of the previous studies were obtained retrospectively from clinical and health insurance databases. The current study is however, one of the few prospective studies involving both private and public doctors. This is also the only longitudinal study on incidence of genital warts among Chinese general population. With the different data collection methods involved in different studies, direct comparisons should however, be made with caution.

A true population census of genital warts incidence is extremely difficult because of the variable spectrum of disease, patient embarrassment in presenting for treatment or self reporting to a survey, the transient nature of the disease and variety of health specialists from whom patients may seek assistance (including pharmacists, alternative/traditional medicine practitioners in addition to physicians). Whether or not attempting a true population census, or representative sample, is worthwhile given the barriers really depends on the purpose for collecting the information (e.g. planning treatment services vs. burden of disease data for cost effectiveness analysis vs. vaccine monitoring). In relation to the latter, one extremely useful outcome of the present study could be to identify enthusiastic practitioners who see young patients presenting with warts who could form the basis of an ongoing surveillance system to monitor trends in presentations with warts at their practices following the introduction of quadrivalent HPV vaccine to the population. This would be more efficient than attempting to repeat the survey over a large number of variably participating practices over time.

In many countries, including the United Kingdom, Italy, Portugal and China, STD surveillance data were obtained from case-reporting made by governmental departments[12,25]. Our results suggested that those systems relying only on data obtained from public clinics are likely to under-estimate the incidence of genital warts. In Hong Kong only 13.1% of such new cases would seek consultation from public doctors. The study demonstrates the feasibility of launching periodic surveillance on genital warts both in the private and public sectors in Hong Kong. It is recommended that similar exercises should be repeated every two to three years so that trends of the disease could be traced.

The findings that males in Hong Kong had higher incidence of genital warts than females were consistent with the high prevalence of risk behaviours observed in the male general population[26]. Two types of HPV vaccine is currently available,[27,28] and quadrivalent vaccine's efficacy in protecting against infection and disease caused by targeted HPV types has been established both for men and women[29,30]. This study hence gives an important piece of baseline information about the epidemiological burden of genital warts, which can be used for evaluating the impact of future vaccination promotion programs at a population level.

The study has several limitations. First, the response rates of the O&G and D&V doctors were somehow disappointing in this study and better collaboration with relevant professional associations is required for future studies of the type. Similarly, the overall response rate for the three types of private doctors was only 69%. We therefore have to assume that the number of new genital wart cases seen by the participating doctors was a representative sample of all such patients in Hong Kong. Although the gender, type and clinic location of the participating doctors was comparable to those doctors who did not participate in this study, the aforementioned assumption could still be invalid. We speculate that participating doctors may be those who have more interest or expertise in genital warts and who may therefore see more cases. In addition, the study sample was based on responders to a 2007 study, newly graduated doctors (since 2007) would not be included in the sample. Second, the data collection period (two weeks) was relatively short, due to the limit of funding the resources. The numbers of patients presenting the genital warts symptoms might be biased because of seasonal change or major festival activities. There may also be a possible increased detection of warts that would otherwise not be diagnosed due to the ongoing prompting of the clinicians through the daily reporting mechanism during the study period. Third, although genital warts are easy to identify by observation, we should admit that it is a limitation to use syndromic management approach in case identification. Lastly, only three categories of private doctors were included into this study whilst other types of specialists may also have managed new genital warts cases during the study period, though such numbers may be relatively small.

## Conclusions

The methodology of the study offers a feasible approach for setting up a surveillance system by including data from both private and public practitioners. The data have important policy implications for prevention and control of genital warts in Hong Kong. It is necessary that both public and private doctors should participate in the STD surveillance in Hong Kong.

## Competing interests

The authors declare that they have no competing interests.

## Authors' contributions

JTFL designed and supervised the study. CLi was the leading author for the paper. M-CLa contributed to the field work and performed statistical analysis. KMH and KKL provided logistic support and assisted in the data collection in the public sectors. HYT coordinated the overall implementation of the study. All authors contributed to the manuscript preparation.

## Pre-publication history

The pre-publication history for this paper can be accessed here:

http://www.biomedcentral.com/1471-2334/10/272/prepub
